# Supplementary material for: The active time model of concurrent choice
Source: PLoS One. 2024 May 21;19(5):e0301173. doi: 10.1371/journal.pone.0301173 (PMC11108226; doi:10.1371/journal.pone.0301173)
Supplement: Attachment — Submitted filename: Response to Reviewers.docx [file pone.0301173.s001.docx]

Dr. J. Mark Cleaveland

Department of Psychological Science

Program in Neuroscience & Behavior

Vassar College, Box 298

124 Raymond Avenue

Poughkeepsie, NY

12604-0298

December 22, 2023

Dear Editors of *PLOS One*,

The following is my response to the reviewer comments regarding my manuscript, “The active time model of concurrent choice.” I would like to thank the reviewers for ploughing through the paper! I would be the first to say that it is not an easy paper. In the manuscript I attempted to construct a model, ATM, for operant choice experiments (specifically, concurrent VI VI) from empirical “first principals,” and then to compare it with three other models. Simultaneously, the manuscript argues that to a large extent, experimental contingencies select for the operative decision rules that an organism brings to bear in an environment. In that sense, no model is “the one to rule them all,” to use a corny Lord of the Rings reference. So again, I am grateful for the reviewers’ willingness to take on the paper and for their useful comments. What follows are how I addressed the their main points.

1. Clarity of prose. Reviewer 1 writes that “there are a lot of trees blocking the readers’ view of the forest,” and “I think that the argument is great, but am overwhelmed by the details.” Reviewer 2 had less of issue with the prose. Nonetheless, I rewrote and edited sections of the first-half of the paper. I believe that it reads somewhat better. I contemplated removing the walk-through of semi-Markov processes, but ultimately decided to leave it, as I believe it is important for understanding the model. Too, my sense is that this isn’t where the paper bogs down. It is in the empirical support that I provided for ATMs core assumptions. I tried moving this to the discussion, but felt that this made the manuscript disjointed. In any event, I took Reviewer 1’s struggles with the prose seriously in my editing.
2. M-value. Reviewer 2 explicitly points out that the description of this metric should be positioned closer in the paper to the simulated data. In the original version, Table 1 contained this data, and it was positioned toward the start of the paper. Reviewer 1 also alluded to this problem in commenting, “Table 1, for instance, is important; but I am not quite sure how to read it.” The problem was that this table was trying to do too much. Therefore, I split it up. The new manuscript has a Table 1 that is restricted to one set of data (choice proportions), and a new Table 2 that is restricted to another data set (M-values). This allowed me to heed Reviewer 2’s advice of positioning the latter data closer in the paper to where the metric is described and discussed.
3. Degrees of freedom. Both reviewers express some unease with ATMs degrees of freedom, and Reviewer 2 suggests that this issue “should [be elaborated] in the discussion.” I do think it is debatable that ATM has more degrees of freedom than the other 3 models considered in the manuscript. Yes, ATM requires switch functions that derived from real data. However, in the paper a single, average function was used for most of the simulations. Further, let us suppose that a future paper were able to characterize a learning mechanism that led to ATM’s switch functions. This, then, would be the true parameter space for the model. Until that time, ATM does require empirically derived switch functions for its predictions, and its true degrees of freedom remains unknown.

However, I also think that the three comparison models considered in the manuscript – momentary maximizing, SET*, and melioration – are being given a pass by the reviewers. I make this point in the revised version of the discussion. SET*, for example, assumes a function that describes the accumulation of interreinforcement distributions in memory. It assumes a separate function for the sampling of these distributions. Each function is uniquely linked to a discriminative stimulus. Finally, in the version considered in the manuscript, another function is assumed to bias the rate of sampling… and this sampling of memory distributions might or might not map on to actual observed behavior. That’s a lot of parameters! Or take momentary maximization. This mechanism states that choice is simply determined by choosing the alternative that has the highest probability of reinforcement. In concurrent VI VI schedules, this means that each discriminative stimulus would be associated with a switch function that relates interresponse times with reinforcement probabilities. This actually sounds a bit like ATM! …except ATM has empirical support for its switch functions, and momentary maximization is explicitly NOT supported as far as its switch functions go. Too, it too does not actually specify how reinforcement probabilities are mapped on to interresponse times, just that they are. I could go on, but the point is that simple-sounding models can have quite a few parameters hiding in the assumptions.

4. Use of AIC. Related to #3, when I wrote the first version of the paper, I did consider providing a more statistically rigorous comparison of the models that were used in the paper. I opted not to do this. First, that would require submitting to a more specialized journal. Second, I honestly wanted to leave the reader considering a more general point – that experimental contingencies to some extent determine choice mechanisms that an organism brings to bear. In this sense there is no “best” model. Or rather, the best model will be prescribed by a particular set of experimental contingencies. It is for this reason that so much attention was paid to undergirding empirically ATM’s core assumptions.

I believe that the above captures the main issues that were brought up by the two reviewers. Reviewer 1 expressed a qualified yes for publication, and I hope that the changes I have made tilt this to a firmer direction. Reviewer 2 was already inclined to accept the manuscript, and I hope that my changes did cause them to move in the other direction!

As I mentioned in my cover letter, I feel that the paper, aside from describing an interesting steady-state model, sets up a strong foundation for follow-up work. For instance, it provides a framework for considering how interoceptive cues (i.e., those generated by the organism rather than the external environment) might come to control behavior. The interoceptive cue utilized by the Active Time model are interresposne times.

Sincerely,

Mark
